# I Am My Peers: How Social Ties Influence E-Cigarette Attitudes, Policy Support, and Use

**DOI:** 10.31586/ojp.2025.6043

**Published:** 2025-03-22

**Authors:** Shervin Assari, Mohammad Mohammadi, Mohammad Pashmchi, Fatemeh Aghaeimeybodi, John Ashley Pallera

**Affiliations:** 1 Department of Internal Medicine, College of Medicine, Charles R. Drew University of Medicine and Science, Los Angeles, USA; 2 School of Medicine, Shahid Sadoughi University of Medical Sciences, Yazd, Iran; 3 Department of Internal Medicine, Shahid Sadoughi University of Medical Sciences, Yazd, Iran; 4 College of Medicine, Charles R. Drew University of Medicine and Science, Los Angeles, USA

**Keywords:** Electronic Cigarettes, Social Influence, Peer Effects, Family Influence, College and University Students, Smoking Behavior

## Abstract

**Background:**

Electronic cigarette (e-cigarette) use is increasingly prevalent among youth and young adults, particularly college and university students. This is a population for whom e-cigarette use is not recommended due to potential health risks, including nicotine addiction and long-term respiratory effects. Social networks play a crucial role in shaping attitudes toward e-cigarettes and influencing use behaviors. However, the relative influence of different social ties—parents, siblings, and friends—on e-cigarette attitudes and use remains unclear.

**Objective:**

This study utilizes data from the SMOKES study to compare the influence of e-cigarette use within different social network sections—parents, friends, and siblings—on personal e-cigarette attitudes and use among college and university students.

**Methods:**

Using a cross-sectional survey of college and university students, we examined the associations between e-cigarette use within different social networks and individual e-cigarette attitudes and use. Multivariate regression models assessed the strength of these associations, adjusting for key demographic and behavioral covariates.

**Results:**

Findings indicate that among college and university students, the strongest influence on both e-cigarette attitudes and use comes from friends who use e-cigarettes. In contrast, parental and sibling e-cigarette use showed weak or non-significant effects. These results suggest that peer influence, rather than family influence, plays a dominant role in shaping e-cigarette-related behaviors and perceptions in young adults.

**Conclusion:**

This study underscores the importance of peer influence in e-cigarette uptake and attitudes among college and university students. Public health interventions aimed at reducing e-cigarette use in this population should consider targeting peer networks rather than focusing solely on family-based influences.

## 1. Introduction

E-cigarette use has rapidly increased among young adults, particularly college and university students, over the past decade [1,2]. While initially marketed as a safer alternative to traditional cigarettes, emerging evidence suggests that e-cigarettes pose their own health risks, including nicotine addiction [3,4], cardiovascular concerns, and respiratory issues. College and university students are at a critical stage of development, where substance use behaviors can have long-term implications for health [5].

Despite public health efforts to discourage e-cigarette use in this age group [6], many students perceive vaping as a socially acceptable behavior, influenced by their immediate social environment. Given that college and university students are legally adults but still in a transitional phase between adolescence and full independence [7], it is crucial to understand the role of social networks in shaping their attitudes and behaviors regarding e-cigarettes [8,9].

Social networks play a significant role in shaping health behaviors, particularly in young adulthood when peer influence is at its peak [10–12]. College and university students often experience shifts in their social environments, moving away from direct parental oversight and becoming more immersed in peer-based interactions [13,14]. As a result, social learning and modeling have become powerful mechanisms for behavior adoption [15–17]. The extent to which different sections of a student’s social network—parents, siblings, and friends—influence their e-cigarette attitudes and use has not been fully explored [18–21]. Understanding these dynamics is essential for designing effective public health interventions aimed at reducing e-cigarette use among college and university students [22–24].

Historically, research on smoking behaviors has emphasized the influence of family members, particularly parents, in shaping children’s attitudes toward tobacco use [25,26]. Parental smoking has been associated with a higher likelihood of smoking initiation among offspring, often through direct exposure and normalization of tobacco use. Siblings may also serve as role models, particularly in families where older siblings engage in smoking or vaping behaviors. However, as young adults enter college, peer influence often surpasses familial influence. College and university students spend more time with their friends than with their families, and social acceptance within peer groups becomes a key motivator for behavioral adoption. While familial influence may remain relevant, particularly in early adolescence, the extent to which it continues to shape e-cigarette use in young adulthood requires further investigation.

Several theoretical frameworks help explain how social relationships influence health behaviors, including e-cigarette use. Social Cognitive Theory (Bandura) [27,28] suggests that behaviors are learned through observation, modeling, and reinforcement within social networks. This means that students who see their friends using e-cigarettes are more likely to adopt the behavior themselves. Social Support Theory [29–32] posits that individuals seek validation and approval from their social circles, making peer acceptance a crucial determinant of health-related choices. The Theory of Planned Behavior emphasizes the role of perceived norms and attitudes in shaping behavior, suggesting that if vaping is normalized within a peer group, individuals are more likely to engage in it [33,34]. These theories highlight the complex interplay of social influence on e-cigarette attitudes and use, warranting a closer examination of which social ties exert the strongest influence.

While extensive research has examined the influence of social networks on traditional cigarette smoking, relatively few studies have explored these influences in the context of e-cigarette use. E-cigarettes differ from conventional cigarettes in multiple ways, including their perceived safety, patterns of use, and social acceptability. Furthermore, prior studies have primarily focused on adolescent populations, leaving a gap in our understanding of how social influences shape e-cigarette behaviors among college and university students. Additionally, few studies have directly compared the relative influence of different social network sections—parents, siblings, and friends—on both attitudes toward e-cigarettes and actual use behaviors.

### Objectives

This study seeks to address these gaps by examining the extent to which parental, sibling, and peer e-cigarette use influences college and university students’ attitudes toward e-cigarettes and their likelihood of using them. Specifically, we aim to: 1) Assess the relative influence of parents, siblings, and friends on college and university students’ e-cigarette use and attitudes, and 2) Determine which social network section has the strongest impact on shaping these behaviors. We hypothesize that among college and university students, friends’ e-cigarette use will have a significantly stronger effect on students’ e-cigarette attitudes and use compared to parental or sibling influence.

Understanding the social dynamics behind e-cigarette use is crucial for developing targeted prevention and intervention strategies. If peer influence is the primary driver of e-cigarette uptake, then public health campaigns should focus on altering peer norms and perceptions about vaping rather than solely targeting family-based interventions. Findings from this study can inform policies aimed at reducing e-cigarette use among young adults, particularly within college and university settings. By identifying the dominant sources of influence, health educators and policymakers can better tailor efforts to curb the rising prevalence of vaping in this vulnerable population.

## 2. Methods

### 2.1. Study Design and Setting

This research is a multi-center, cross-sectional study examining attitudes, knowledge, and behaviors related to electronic cigarettes and other tobacco products among university students in Iran. The study, known as the SMOKES study (Study of Measurement of Knowledge and Examination of Support for Tobacco Control Policies) [35,36], was conducted between November 2024 and January 2025 across multiple higher education institutions in the country.

### 2.2. Ethical Considerations

The SMOKES study adhered to the ethical guidelines established by the Declaration of Helsinki. Ethical approval was granted by the Ethics Committee of Shahid Sadoughi University of Medical Sciences, Yazd, Iran (Ethics Code: IR.SSU.MEDICINE.REC.1403.159). All participants provided informed consent before participating in the study, and participation was entirely voluntary.

### 2.3. Overview of the SMOKES Study

The SMOKES study [35,36] was designed to assess university students’ perspectives on tobacco control measures, with a particular focus on their level of support for such policies. In addition to policy attitudes, the study examined patterns of tobacco use, including electronic cigarette consumption. Data were collected on students’ knowledge, attitudes, and motivations regarding tobacco use, as well as the broader social and contextual factors influencing their behaviors. This comprehensive approach aimed to explore how personal tobacco use influences support for regulatory policies. The insights derived from this study can inform policy development and targeted public health interventions aimed at reducing tobacco-related harm. Further details regarding the study’s methodology, rationale, and sample characteristics have been previously published.

### 2.4. Sampling Strategy

To ensure diversity in terms of ethnicity, geographic distribution, and academic disciplines, the study included universities from 15 different provinces across Iran, selecting at least one higher education institution per province. While efforts were made to reflect the demographic composition of Iranian university students, the findings may not be fully generalizable to the entire student population at a national level.

### 2.5. Data Collection and Survey Instrument

The study employed an online survey, administered in Farsi, to collect data across multiple domains. The survey link was disseminated through social media platforms and student groups, inviting students to voluntarily participate. The questionnaire covered a range of topics, including demographic characteristics, tobacco use behaviors, and attitudes toward tobacco control policies.

### 2.6. Study Measures

#### Demographic and Socioeconomic Data:

Participants provided information on key demographic and socioeconomic indicators, including ethnicity, family and personal income, marital status, employment status, and place of residence. Additionally, academic-related variables such as province of study, major, level of education (ranging from associate to doctoral degrees), type of institution, and year of study were collected.

#### Electronic Cigarette Use:

Participants reported their history of electronic cigarette use, distinguishing between ever-use and current-use. The frequency of use was also recorded. While conventional cigarette and hookah use were assessed, these variables were not included in the primary analysis, as the focus was on electronic cigarette use. Each tobacco product was assessed using single-item questions to determine both lifetime and current use.

#### Attitudes Toward Electronic Cigarettes:

Attitudes toward electronic cigarettes were measured using an eight-item scale assessing participants’ perceptions. Statements such as “Using electronic cigarettes is enjoyable” were rated on a five-point Likert scale, ranging from 1 (Strongly Disagree) to 5 (Strongly Agree). A composite score was calculated to represent pro-electronic cigarette attitudes, with higher scores indicating more favorable views of e-cigarettes.

#### Support for Electronic Cigarette Policies:

To evaluate support for tobacco control policies, participants responded to several items regarding regulations on electronic cigarette advertising, sales, and use. For example, one item asked, “To what extent do you support banning the advertisement and sale of electronic cigarettes on online platforms such as Instagram?” Responses were recorded using a five-point Likert scale (1 = Strongly Disagree to 5 = Strongly Agree), and an overall policy support score was computed by averaging responses. Higher scores reflected stronger endorsement of tobacco control measures.

### 2.7. Statistical Analysis

Data were analyzed using Stata 18. Descriptive statistics were calculated to summarize the sample characteristics, with categorical variables presented as frequencies and percentages and continuous variables as means with standard deviations. Composite scores were created for tobacco control policy support, and a linear regression model (ordinary least squares) was conducted to examine the association between tobacco use and policy support, setting statistical significance at p < 0.05. To further analyze the relationships among knowledge, attitudes, and behavior, a structured approach incorporating confirmatory factor analysis (CFA) and structural equation modeling (SEM) was employed. Descriptive analyses included t-tests for continuous variables and chi-square tests for categorical variables to assess differences in electronic cigarette use across demographic subgroups. A structural equation model (SEM) was utilized to investigate the associations between knowledge of electronic cigarettes, attitudes toward vaping, and usage patterns, while controlling for demographic factors such as age, sex, ethnicity, and family income. The analysis explored both direct and indirect pathways, assessing whether attitudes mediated the relationship between knowledge and e-cigarette use. Mediation analysis was conducted by estimating indirect and total effects within the SEM framework. Model fit was assessed using standard goodness-of-fit indices, including the Comparative Fit Index (CFI), Tucker-Lewis Index (TLI), and Root Mean Square Error of Approximation (RMSEA). Fit was considered acceptable if CFI and TLI exceeded 0.90 and RMSEA and SRMR were below 0.08. To enhance measurement accuracy, latent variables were constructed for key constructs—knowledge, attitudes, and behaviors—using CFA. Latent variables were inferred from multiple observed indicators, reducing measurement error and improving construct validity. For instance, knowledge was measured using multiple survey items assessing factual understanding, attitudes were derived from responses reflecting perceptions of e-cigarette risks and benefits, and behaviors were assessed through self-reported electronic cigarette use patterns. The incorporation of latent variables allowed for a more precise estimation of the relationships among these constructs, providing a deeper understanding of the factors influencing vaping behaviors among university students. This methodological approach strengthens the reliability and validity of the study findings and offers valuable insights for public health interventions and tobacco control policies.

## 3. Results

A total of 2,403 university and college and university students participated in this study, with an equal representation of male and female students (1:1 ratio). The average age of participants was 22.30 years (SE = 0.07; 95% CI = 22.16 to 22.44), with ages ranging from 15 to 60 years. A detailed presentation of the participant distribution by academic level, academic subject, geographic representation, ethnic makeup, and institution type is provided in Table 1.

Table 2 shows the results of the structural equation modeling (SEM) analysis indicate several significant associations between tobacco use by family members and peers, demographic factors, and three key outcomes: pro e-cigarette attitude, policy support to ban e-cigarettes, and e-cigarette use.

For pro e-cigarette attitude, tobacco use by friends was a strong and significant predictor (B = 0.225, SE = 0.022, p < 0.001), while tobacco use by siblings also showed a significant positive association (B = 0.044, SE = 0.021, p = 0.039). In contrast, tobacco use by fathers (B = 0.029, SE = 0.021, p = 0.176) and mothers (B = 0.018, SE = 0.021, p = 0.399) was not significantly associated. Older age was linked to a greater pro e-cigarette attitude (B = 0.099, SE = 0.015, p < 0.001), whereas being an ethnic minority (B = −0.086, SE = 0.021, p < 0.001) and female (B = −0.138, SE = 0.022, p < 0.001) were associated with lower pro e-cigarette attitudes. Family income did not show significant effects on pro e-cigarette attitudes (B = 0.036, SE = 0.022, p = 0.105).

Regarding policy support for banning e-cigarettes, tobacco use by friends was significantly associated with lower support (B = −0.175, SE = 0.022, p < 0.001), whereas tobacco use by fathers (B = −0.019, SE = 0.022, p = 0.389), mothers (B = −0.028, SE = 0.021, p = 0.189), and siblings (B = −0.024, SE = 0.022, p = 0.268) did not show significant associations. Older age was linked to greater support for a ban (B = 0.043, SE = 0.017, p = 0.011), as was being an ethnic minority (B = 0.050, SE = 0.022, p = 0.022) and female (B = 0.046, SE = 0.022, p = 0.041). Family income, however, was negatively associated with policy support (B = −0.050, SE = 0.022, p = 0.026), suggesting that higher-income individuals were less likely to support a ban.

For e-cigarette use, tobacco use by friends was the strongest predictor (B = 0.302, SE = 0.018, p < 0.001), followed by tobacco use by siblings (B = 0.053, SE = 0.019, p = 0.005) and fathers (B = 0.049, SE = 0.019, p = 0.009). The association between e-cigarette use and maternal tobacco use was not statistically significant (B = 0.032, SE = 0.019, p = 0.083). Older individuals were more likely to use e-cigarettes (B = 0.045, SE = 0.006, p < 0.001), as were those with higher family income (B = 0.068, SE = 0.019, p < 0.001). Ethnic minority status was marginally associated with lower e-cigarette use (B = −0.035, SE = 0.018, p = 0.058), whereas females were significantly less likely to use e-cigarettes (B = −0.122, SE = 0.019, p < 0.001).

## 4. Discussion

This study aimed to examine the relative influence of different social network sections—parents, siblings, and friends—on college and university students’ e-cigarette attitudes and use. Given the well-documented role of social influence in substance use behaviors, we hypothesized that friends’ e-cigarette use would exert a significantly stronger impact on students’ attitudes and behaviors compared to parental or sibling influence. This hypothesis was based on the understanding that as young adults transition to college life, peer networks become more influential than family in shaping behaviors, including substance use.

Our findings supported this hypothesis, demonstrating that among college and university students, friends’ e-cigarette use is the strongest predictor of both e-cigarette attitudes and personal use. While parental and sibling e-cigarette use was also assessed, their influence was weak or non-significant. Similarly, a study found that Romanian university students who had experimented with e-cigarettes were significantly more likely to report having friends (84.4%) and colleagues (62.3%) who also used e-cigarettes, compared to only 4.2% who reported parental use [55]. These results suggest that familial influence diminishes in young adulthood, while peer influence remains a dominant force. This aligns with prior research on smoking behaviors and extends the understanding to e-cigarette use, which has unique social and cultural dynamics.

For adolescents, research indicates that exposure to smoking among family members and peers is a significant predictor of smoking initiation. However, the extent to which this influence extends to electronic cigarette (e-cigarette) use has not been comprehensively analyzed. A systematic review and meta-analysis were conducted using studies retrieved from PubMed and ScienceDirect up to December 2016. A random-effects model was applied to calculate summary odds ratios (ORs) with 95% confidence intervals (CIs). The analysis included 21 studies and found a positive association between adolescent e-cigarette use and smoking behavior among both family members (OR=1.47, 95% CI=1.30–1.66) and peers (OR=2.72, 95% CI=1.87–3.95), even after accounting for the adolescent’s own smoking status. When examining specific family relationships, the influence of sibling smoking (OR=1.87, 95% CI=1.35–2.60) appeared stronger compared to parental smoking (OR=1.41, 95% CI=1.19–1.68) and smoking by other relatives (OR=1.39, 95% CI=1.12–1.72) [37].

Our findings align with Bandura’s Social Cognitive Theory, [27] which suggests that behaviors are learned through observation, modeling, and reinforcement. College and university students who see their friends using e-cigarettes are likely to adopt similar behaviors due to repeated exposure and perceived social acceptance. The limited influence from parents or siblings suggests that observational learning is more powerful in peer contexts, particularly in young adulthood. Wallace and Roche (2018) investigated the relationship between e-cigarette use, social status, and peer influence among college students (n=175) [56]. Their findings indicated that each additional friend who used e-cigarettes was associated with a 2.44-fold increase (95% CI [1.10, 5.40]) in the odds of having accepted a past e-cigarette offer from a friend and a 2.33-fold increase (95% CI [1.58, 3.56]) in the odds of being willing to accept such an offer in the future. Additionally, students who perceived e-cigarettes as socially beneficial had 2.2 times greater odds (95% CI [1.19, 4.33]) of accepting a future offer [56].

Social Support Theory, as developed by James House and others, [38,39] posits that individuals seek various types of social support, often from those similar to them. This theory highlights the tendency for like-minded individuals to gravitate toward each other, seeking validation and reinforcement within their peer groups. College and university students are in a phase of life where independence from family is emphasized, and peer acceptance becomes a primary source of emotional and behavioral reinforcement. In a study of 387 NCAA student-athletes across 23 teams, it was found that athletes who perceived higher levels of peer acceptance within their team reported significantly more permissive attitudes toward alcohol use (b = 0.20, p < .05) [57]. These findings suggest that students who feel more socially accepted may be more likely to engage in behaviors endorsed by their peer group, such as alcohol consumption, in order to maintain or reinforce their sense of belonging [57]. The clustering of attitudes within teams further supports the idea that social norms and behaviors are shaped by peer dynamics rather than familial influence—highlighting the powerful role of peer-based social reinforcement in emerging adulthood [57]. While this study provides valuable insight into the role of peer-based social reinforcement, its generalizability is limited by its exclusive focus on student-athletes and its omission of other substances like tobacco or e-cigarettes. That said, given the growing popularity of e-cigarettes among young adults and their common presence in social settings, it is possible that e-cigarette use may be perceived by college students as more analogous to alcohol use than to traditional tobacco products like cigarettes [58].

Social Network and Graph Theory further explains how behaviors spread through network connections, with peer groups acting as the most immediate and impactful “nodes” of influence [40,41]. This network-driven adoption of e-cigarette use is particularly relevant in environments where vaping is normalized. In a review of 13 studies looking at social network factors and addictive behaviors among college students, it was found that individuals embedded in more interconnected and centralized networks were significantly more likely to engage in substance use, particularly alcohol [59]. The review highlighted that key network characteristics—such as density, centrality, and reciprocated ties—were positively associated with higher levels of risky behaviors [58]. These findings support the idea that vaping may spread not only through curiosity or individual decision-making, but through social exposure and reinforcement within tightly knit peer groups—particularly in settings like college campuses, where peer influence is highly concentrated and dynamic.

The Theory of Planned Behavior suggests subjective norms, attitudes, and perceived behavioral control shape individual behaviors [33,34]. Subjective norms refer to the perception of a behavior’s acceptability within a social network [42]. In a study examining the influence of descriptive and injunctive norms on e-cigarette use among college students (n = 138; 72% female) at a university in the northeastern United States, both types of norms were found to independently predict past 30-day e-cigarette use [60]. This indicates that students are influenced not only by how common vaping is among their peers (descriptive norms), but also by the extent to which their peers approve of the behavior (injunctive norms). In line with these findings, our results further demonstrate that subjective norms within peer groups—rather than those within families—are the primary drivers of e-cigarette use among college and university students. If students perceive vaping as an accepted behavior among their peers, they are more likely to adopt it, regardless of parental attitudes or family background.

### 4.1. Implications

Peer influence plays a crucial role in shaping health behaviors, particularly among college and university students, making it essential to incorporate peer support and peer-based interventions in efforts to reduce e-cigarette use [37]. Young adults are highly responsive to their peers’ behaviors and attitudes, often looking to social groups for cues on what is acceptable or desirable.

Interventions should engage peer leaders and supportive social circles to leverage social reinforcement for promoting healthier behaviors. Peer-based interventions, such as mentorship programs, peer-led educational workshops, and social media campaigns featuring influential students, can help create environments where non-use is the norm. These strategies not only offer credible, relatable sources of information but also provide emotional and social support that empowers students to resist social pressures to vape.

Another critical aspect of peer influence is the misperception of substance use prevalence among social groups. Many young adults overestimate how common e-cigarette use is within their networks, believing that a majority of their peers engage in vaping even when actual usage rates are much lower. When students assume that e-cigarette use is the norm, they may feel pressured to adopt the behavior to fit in, even in environments where the majority of students do not vape. Addressing these misperceptions through social norm campaigns can be a key intervention strategy. By providing accurate data on peer substance use, public health initiatives can reduce the perceived social pressure to vape, reinforcing the reality that e-cigarette use is not as widespread or necessary for social belonging as some students might assume.

Given the strong role of peer influence in e-cigarette use among college and university students, policies aimed at reducing vaping in this population should prioritize strategies that target peer networks. Traditional family-centered interventions may be less effective for this demographic. For younger populations, such as early or mid-adolescents, family interventions may still play a significant role. For college and university students, policies should focus on altering perceived or actual social norms surrounding vaping. Strategies may include restricting e-cigarette marketing and advertisement, limiting the public display or use of e-cigarettes, implementing campus-wide bans, and promoting peer-led anti-vaping campaigns [43–46]. These measures can help disrupt the normalization of vaping in college settings. Health screenings for e-cigarette use in college populations should incorporate social network assessments [47]. Instead of solely asking about personal use, clinicians and public health professionals should screen for exposure to e-cigarette use within peer networks. Understanding the social context of e-cigarette use can help healthcare providers identify at-risk students and offer targeted interventions that address peer influence.

Social network interventions [48–52] should focus on altering social structures and disrupting the transmission of pro-vaping norms. Given our findings, interventions should target key nodes of influence by identifying and engaging influential peer leaders who can advocate for anti-vaping norms. Additionally, leveraging social media platforms is essential [47,53,54], as much of college and university students’ social interactions occur online. Digital platforms offer a powerful space for disseminating anti-vaping messages. Finally, creating counter-narratives through compelling, youth-oriented campaigns can highlight the risks of e-cigarette use while promoting healthier alternatives, shifting social perceptions away from vaping.

### 4.2. Limitations

Despite its strengths, this study has several limitations. First, its cross-sectional design prevents causal inferences. We cannot determine whether peer influence causes e-cigarette use, if individuals are predisposed to vaping self-select into vaping peer groups, or if individuals who use e-cigarettes develop a belief that their peers vape to avoid cognitive dissonance. Second, the study relied on self-reported data, which may be subject to social desirability bias. Additionally, variation in associations was not examined based on ethnicity, gender, geographic location, or type of e-cigarette device used. Future studies incorporating biomarkers and nicotine tests could enhance the validity of findings. Third, the generalizability of findings may be limited to college and university students, necessitating further research on non-college young adults.

### 4.3. Strengths

Despite these limitations, the study has several strengths. The SMOKES study[35,36] is a well-established dataset with robust measures of e-cigarette knowledge, attitudes, policy support, and behaviors. The study contributes novel insights by directly comparing the relative influence of parents, siblings, and friends—an area with limited prior research in this age group. Additionally, the findings are highly relevant for informing interventions targeted at diverse college and university students, a key demographic in public health efforts to reduce vaping.

### 4.4. Future research

Future research should focus on designing and testing interventions that account for the dominant role of peer influence in young adult vaping behaviors. One important area for further study is the development of peer-based intervention programs, examining whether peer-led health educators can effectively reduce e-cigarette use. Another key area is evaluating the effects of policies and interventions by investigating how strategies such as advertising restrictions and campus bans impact students based on their social network structures. Additionally, research should explore whether altering beliefs of key social network influencers [9]can shift vaping behaviors at a larger scale. Finally, longitudinal studies are needed to track changes in e-cigarette use patterns over time and assess the evolving influence of peers and family, allowing for stronger causal interpretations and more effective intervention strategies.

## 5. Conclusion

This study highlights the stronger role of peer influence compared to family in shaping e-cigarette attitudes and behaviors among college and university students. Given that perceived peer vaping significantly predicts both attitudes and use, interventions should prioritize peer-driven strategies over family-based approaches. Public health efforts should leverage social networks, digital platforms, and peer influencers [9] to create environments where vaping is less socially acceptable. Addressing the social drivers of e-cigarette use is essential for reducing its prevalence in college populations and preventing long-term health consequences.

## Figures and Tables

**Figure 1.**
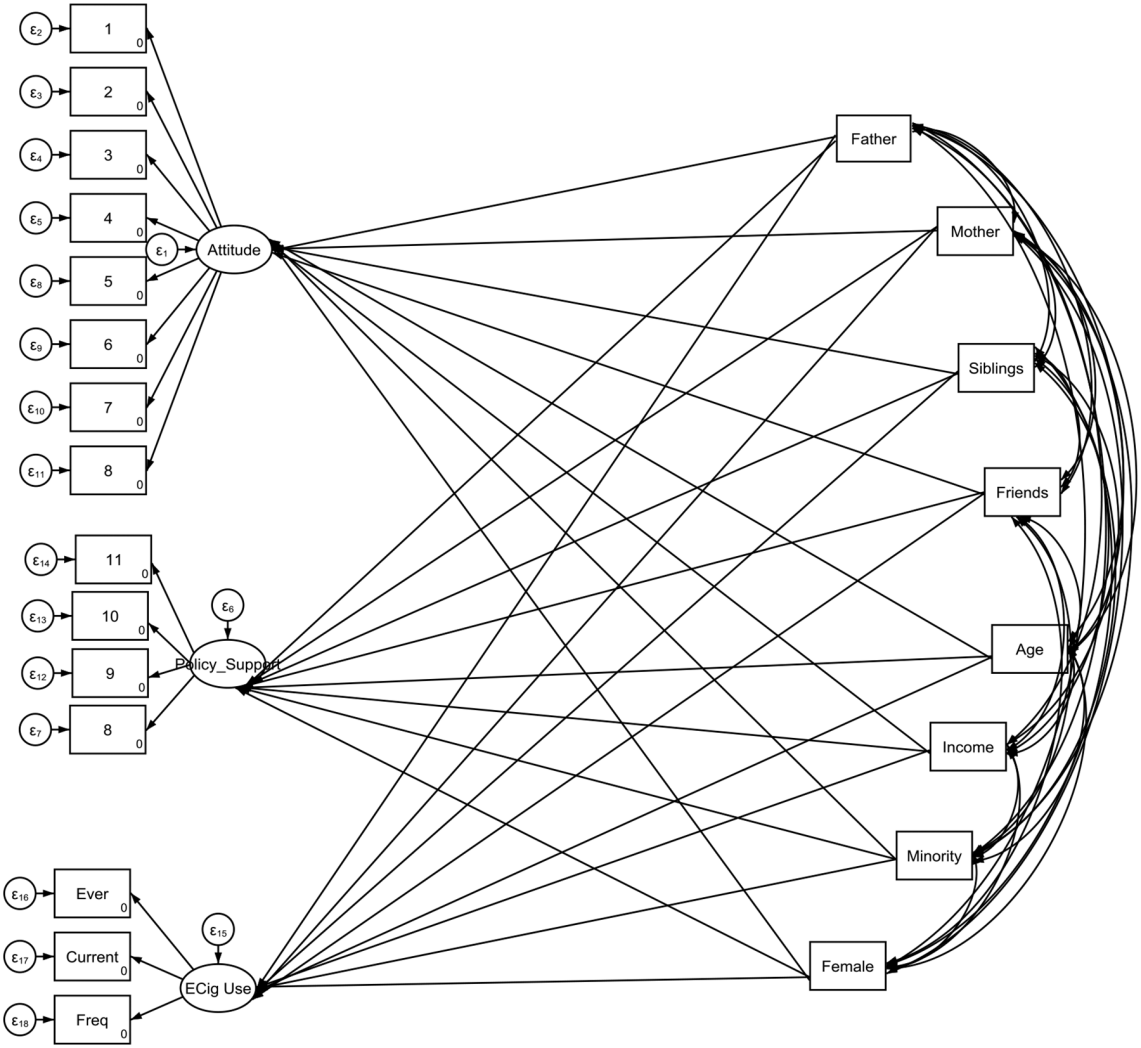
SEM

**Table 1.** Participants in the Study

	n	%
University Major		
Humanities and Social Sciences	233	9.76
Basic Sciences	90	3.77
Engineering and Technology Sciences	318	13.32
Medicine	1,036	43.38
Dentistry	212	8.88
Pharmacy	68	2.85
Nursing	118	4.94
Allied Health	194	8.12
Agriculture and Natural Resources	11	0.46
Architecture and Arts	88	3.69
Veterinary Medicine	20	0.84
Province		
Tehran	255	10.64
Khorasan Razavi	210	8.76
Fars	136	5.68
East Azerbaijan	135	5.63
Esfahan	221	9.22
Yazd	350	14.61
Khouzestan	119	4.97
Kerman	119	4.97
Sistan and Balouchestan	118	4.92
Kermanshah	166	6.93
Mazandaran	262	10.93
Gilan	138	5.76
Semnan	73	3.05
Alborz	52	2.17
Hamedan	42	1.75
Residence		
Living with Family	1,198	49.9
Dormitory	970	40.4
Private Housing (Without Family)	228	9.5
Other	5	0.21
Sex		
Male	1,201	50
Female	1,201	50
Ethnicity		
Fars	1,356	57.75
Turk	265	11.29
Lur	136	5.79
Kurd	160	6.81
Mazani	138	5.88
Arab	28	1.19
Balouch	47	2
Gilak	112	4.77
Bakhtiari	41	1.75
Semnani	12	0.51
Other	53	2.26
		
University Level		
Associate’s Degree	62	2.58
Bachelor’s Degree	926	38.57
Master’s Degree	160	6.66
Doctorate or Higher	1,253	52.19
University Type		
Governmental	1,826	76.12
Non-Governmental/Private	573	23.88

**Table 2.** Summary of SEM

Outcome	Predictor	B	SE	95%	CI	p
Pro E-Cig Attitude						
	Tobacco Use by Friends	0.225	0.022	0.182	0.268	< 0.001
	Tobacco Use by Father	0.029	0.021	−0.013	0.071	0.176
	Tobacco Use by Mother	0.018	0.021	−0.024	0.060	0.399
	Tobacco Use by Sibling	0.044	0.021	0.002	0.086	0.039
	Age (Yr)	0.099	0.015	0.070	0.129	< 0.001
	Family Income	0.036	0.022	−0.007	0.079	0.105
	Ethnic Minority	−0.086	0.021	−0.128	−0.045	< 0.001
	Sex (Female)	−0.138	0.022	−0.180	−0.095	< 0.001
						
Policy Support to Ban E-Cig						
	Tobacco Use by Friends	−0.175	0.022	−0.218	−0.132	< 0.001
	Tobacco Use by Father	−0.019	0.022	−0.061	0.024	0.389
	Tobacco Use by Mother	−0.028	0.021	−0.070	0.014	0.189
	Tobacco Use by Sibling	−0.024	0.022	−0.066	0.018	0.268
	Age (Yr)	0.043	0.017	0.010	0.076	0.011
	Family Income	−0.050	0.022	−0.094	−0.006	0.026
	Ethnic Minority	0.050	0.022	0.007	0.092	0.022
	Sex (Female)	0.046	0.022	0.002	0.090	0.041
						
E-Cig Use						
	Tobacco Use by Friends	0.302	0.018	0.266	0.338	< 0.001
	Tobacco Use by Father	0.049	0.019	0.012	0.085	0.009
	Tobacco Use by Mother	0.032	0.019	−0.004	0.069	0.083
	Tobacco Use by Sibling	0.053	0.019	0.016	0.090	0.005
	Age (Yr)	0.045	0.006	0.033	0.057	< 0.001
	Family Income	0.068	0.019	0.031	0.104	< 0.001
	Ethnic Minority	−0.035	0.018	−0.071	0.001	0.058
	Sex (Female)	−0.122	0.019	−0.159	−0.085	< 0.001

## References

[1] Chaffee BW Electronic Cigarettes: Trends, Health Effects and Advising Patients Amid Uncertainty. J Calif Dent Assoc 2019, 47, 85–92. 30976150 PMC6454567

[2] Chaffee BW; Couch ET; Gansky SA Trends in characteristics and multi-product use among adolescents who use electronic cigarettes, United States 2011–2015. PLoS One 2017, 12, e0177073, doi:10.1371/journal.pone.0177073. 28475634 PMC5419603

[3] Goldenson NI; Leventhal AM; Stone MD; McConnell RS; Barrington-Trimis JL Associations of Electronic Cigarette Nicotine Concentration With Subsequent Cigarette Smoking and Vaping Levels in Adolescents. JAMA Pediatr 2017, 171, 1192–1199, doi:10.1001/jamapediatrics.2017.3209. 29059261 PMC5779618

[4] Vollstädt-Klein S; Grundinger N; Görig T; Szafran D; Althaus A; Mons U; Schneider S Study protocol: evaluation of the addictive potential of e-cigarettes (EVAPE): neurobiological, sociological, and epidemiological perspectives. BMC Psychol 2021, 9, 181, doi:10.1186/s40359-021-00682-8. 34794514 PMC8600891

[5] Chan GCK; Stjepanović D; Lim C; Sun T; Shanmuga Anandan A; Connor JP; Gartner C; Hall WD; Leung J Gateway or common liability? A systematic review and meta-analysis of studies of adolescent e-cigarette use and future smoking initiation. Addiction 2021, 116, 743–756, doi:10.1111/add.15246. 32888234

[6] Hiler M; Spindle TR; Dick D; Eissenberg T; Breland A; Soule E Reasons for Transition From Electronic Cigarette Use to Cigarette Smoking Among Young Adult College Students. J Adolesc Health 2020, 66, 56–63, doi:10.1016/j.jadohealth.2019.09.003. 31699605 PMC6935466

[7] Roberts G Why we should focus on adolescents and young adults. Clin Exp Allergy 2020, 50, 650–651, doi:10.1111/cea.13666. 32478456

[8] Valente TW; Piombo SE; Edwards KM; Waterman EA; Banyard VL Social Network Influences on Adolescent E-cigarette Use. Subst Use Misuse 2023, 58, 780–786, doi:10.1080/10826084.2023.2188429. 36924165 PMC10112417

[9] Vassey J; Valente T; Barker J; Stanton C; Li D; Laestadius L; Cruz TB; Unger JB E-cigarette brands and social media influencers on Instagram: a social network analysis. Tob Control 2023, 32, e184–e191, doi:10.1136/tobaccocontrol-2021-057053. 35131947 PMC9473311

[10] Reynolds AD; Crea TM Peer influence processes for youth delinquency and depression. J Adolesc 2015, 43, 83–95, doi:10.1016/j.adolescence.2015.05.013. 26066630

[11] Monahan KC; Steinberg L; Cauffman E Affiliation with antisocial peers, susceptibility to peer influence, and antisocial behavior during the transition to adulthood. Dev Psychol 2009, 45, 1520–1530, doi:10.1037/a0017417. 19899911 PMC2886974

[12] Andrews NCZ Prestigious Youth are Leaders but Central Youth are Powerful: What Social Network Position Tells us About Peer Relationships. J Youth Adolesc 2020, 49, 631–644, doi:10.1007/s10964-019-01080-5. 31301026

[13] Negriff S The Influence of Online-Only Friends on the Substance Use of Young Adults with a History of Childhood Maltreatment. Subst Use Misuse 2019, 54, 120–129, doi:10.1080/10826084.2018.1508299. 30372360 PMC6379128

[14] Field NH; Prinstein MJ Reconciling multiple sources of influence: Longitudinal associations among perceived parent, closest friend, and popular peer injunctive norms and adolescent substance use. Child Dev 2023, 94, 809–825, doi:10.1111/cdev.13898. 36779425 PMC10293111

[15] Samek DR; Rueter MA Considerations of elder sibling closeness in predicting younger sibling substance use: social learning versus social bonding explanations. J Fam Psychol 2011, 25, 931–941, doi:10.1037/a0025857. 21988080 PMC3237833

[16] Capaldi DM; Tiberio SS; Kerr DCR Assessing Associations in Substance Use across Three Generations: From Grandparents to Sons and from Sons to Their Children. Contemp Soc Sci 2018, 13, 288–304, doi:10.1080/21582041.2018.1433313. 31435489 PMC6703815

[17] Kothari BH; Sorenson P; Bank L; Snyder J Alcohol and Substance Use in Adolescence and Young Adulthood: The Role of Siblings. J Fam Soc Work 2014, 17, 324–343, doi:10.1080/10522158.2014.924457. 25484550 PMC4256025

[18] Reiner A; Steinhoff P The association of social networks and depression in community-dwelling older adults: a systematic review. Syst Rev 2024, 13, 161, doi:10.1186/s13643-024-02581-6. 38902787 PMC11188217

[19] Bahl NKH; Øversveen E; Brodahl M; Nafstad HE; Blakar RM; Ness O; Landheim AS; Tømmervik K In what ways do emerging adults with substance use problems experience their communities as influencing their personal recovery processes? J Community Psychol 2022, 50, 3070–3100, doi:10.1002/jcop.22816. 35187694 PMC9545888

[20] Moriarity DP; Bart CP; Stumper A; Jones P; Alloy LB Mood symptoms and impairment due to substance use: A network perspective on comorbidity. J Affect Disord 2021, 278, 423–432, doi:10.1016/j.jad.2020.09.086. 33010567 PMC7704896

[21] Hussong AM; Ennett ST; McNeish DM; Cole VT; Gottfredson NC; Rothenberg WA; Faris RW Social network isolation mediates associations between risky symptoms and substance use in the high school transition. Dev Psychopathol 2020, 32, 615–630, doi:10.1017/s095457941900049x. 31232267 PMC7011186

[22] Purcell C; Dibben G; Hilton Boon M; Matthews L; Palmer VJ; Thomson M; Smillie S; Simpson SA; Taylor RS Social network interventions to support cardiac rehabilitation and secondary prevention in the management of people with heart disease. Cochrane Database Syst Rev 2023, 6, Cd013820, doi:10.1002/14651858.CD013820.pub2. PMC10305790 37378598

[23] Dembo RS; Huntington N; Mitra M; Rudolph AE; Lachman ME; Mailick MR Social network typology and health among parents of children with developmental disabilities: Results from a national study of midlife adults. Soc Sci Med 2022, 292, 114623, doi:10.1016/j.socscimed.2021.114623. 34891030 PMC8748422

[24] Li JB; Feng LF; Wu AMS; Mai JC; Chen YX; Mo PKH; Lau JTF Roles of Psychosocial Factors on the Association Between Online Social Networking Use Intensity and Depressive Symptoms Among Adolescents: Prospective Cohort Study. J Med Internet Res 2021, 23, e21316, doi:10.2196/21316. 34546173 PMC8493459

[25] Broun A; Haynie D; Choi K Parental Anti-Smoking Encouragement as a Longitudinal Predictor of Young Adult Cigarette and E-cigarette Use in a US National Study. Nicotine Tob Res 2021, 23, 1468–1474, doi:10.1093/ntr/ntab026. 33592090 PMC8372650

[26] Asawa K; Doshi A; Bhat N; Tak M; Chhajlani A; Bhosle S; Jain S; Shah D Relationship between Parental Bonding and Tobacco Specific Practices as Predictors of Tobacco Usage in Adults. J Clin Diagn Res 2017, 11, Zc36–zc41, doi:10.7860/jcdr/2017/26850.10173. PMC5583935 28893040

[27] Bandura A Social cognitive theory: an agentic perspective. Annu Rev Psychol 2001, 52, 1–26, doi:10.1146/annurev.psych.52.1.1. 11148297

[28] Rinderknecht K; Smith C Social cognitive theory in an after-school nutrition intervention for urban Native American youth. J Nutr Educ Behav 2004, 36, 298–304, doi:10.1016/s1499-4046(06)60398-9. 15617611

[29] Lakey B; Cohen S Social support theory and measurement. 2000.

[30] Christiaen E; Goossens MG; Descamps B; Larsen LE; Boon P; Raedt R; Vanhove C Dynamic functional connectivity and graph theory metrics in a rat model of temporal lobe epilepsy reveal a preference for brain states with a lower functional connectivity, segregation and integration. Neurobiol Dis 2020, 139, 104808, doi:10.1016/j.nbd.2020.104808. 32087287

[31] Farahani FV; Karwowski W; Lighthall NR Application of Graph Theory for Identifying Connectivity Patterns in Human Brain Networks: A Systematic Review. Front Neurosci 2019, 13, 585, doi:10.3389/fnins.2019.00585. 31249501 PMC6582769

[32] Zheng K; Wang H; Li J; Yan B; Liu J; Xi Y; Zhang X; Yin H; Tan Q; Lu H; Structural networks analysis for depression combined with graph theory and the properties of fiber tracts via diffusion tensor imaging. Neurosci Lett 2019, 694, 34–40, doi:10.1016/j.neulet.2018.11.025. 30465819

[33] Guo Q; Johnson CA; Unger JB; Lee L; Xie B; Chou C-P; Palmer PH; Sun P; Gallaher P; Pentz M Utility of the theory of reasoned action and theory of planned behavior for predicting Chinese adolescent smoking. Addictive behaviors 2007, 32, 1066–1081, doi:10.1016/j.addbeh.2006.07.015. 16934414

[34] Topa G; Moriano JA Theory of planned behavior and smoking: Meta-analysis and SEM model. Substance abuse and rehabilitation 2010, 1, 23–33, doi:10.2147/SAR.S15168. 24474850 PMC3819188

[35] Assari S; Mohammadi M; Pashmchi M; Aghaeimeybodi F Tobacco-control policy support and tobacco use: SMOKES study. Global Journal of Epidemiology and Infectious Disease 2025, 5, 6011. 10.31586/gjcd.2025.6005 PMC12140087 40476180

[36] Assari S; Mohammadi M; Pashmchi M; Aghaeimeybodi F SMOKES: Study of Measurement of Knowledge and Examination of Support for tobacco control policies. Global Journal of Cardiovascular Diseases 2025, 4, 6005. 10.31586/gjcd.2025.6005 PMC12140087 40476180

[37] Wang JW; Cao SS; Hu RY Smoking by family members and friends and electronic-cigarette use in adolescence: A systematic review and meta-analysis. Tob Induc Dis 2018, 16, 05, doi:10.18332/tid/84864. 31516405 PMC6659504

[38] Gottlieb BH; Bergen AE Social support concepts and measures. J Psychosom Res 2010, 69, 511–520, doi:10.1016/j.jpsychores.2009.10.001. 20955871

[39] Turner RJ; Marino F Social support and social structure: a descriptive epidemiology. J Health Soc Behav 1994, 35, 193–212. 7983334

[40] Jones PJ; Ma R; McNally RJ Bridge Centrality: A Network Approach to Understanding Comorbidity. Multivariate Behav Res 2021, 56, 353–367, doi:10.1080/00273171.2019.1614898. 31179765

[41] Ucer S; Ozyer T; Alhajj R Explainable artificial intelligence through graph theory by generalized social network analysis-based classifier. Sci Rep 2022, 12, 15210, doi:10.1038/s41598-022-19419-7. 36075941 PMC9458666

[42] Muzaffar H; Chapman-Novakofski K; Castelli DM; Scherer JA The HOT (Healthy Outcome for Teens) project. Using a web-based medium to influence attitude, subjective norm, perceived behavioral control and intention for obesity and type 2 diabetes prevention. Appetite 2014, 72, 82–89, doi:10.1016/j.appet.2013.09.024. 24099704

[43] Silver NA; Bertrand A; Kucherlapaty P; Schillo BA Examining influencer compliance with advertising regulations in branded vaping content on Instagram. Front Public Health 2022, 10, 1001115, doi:10.3389/fpubh.2022.1001115. 36699883 PMC9869128

[44] Pettigrew S; Santos JA; Pinho-Gomes AC; Li Y; Jones A Exposure to e-cigarette advertising and young people’s use of e-cigarettes: A four-country study. Tob Induc Dis 2023, 21, 141, doi:10.18332/tid/172414. 37881174 PMC10594952

[45] Zheng X; Li W; Wong SW; Lin HC Social media and E-cigarette use among US youth: Longitudinal evidence on the role of online advertisement exposure and risk perception. Addict Behav 2021, 119, 106916, doi:10.1016/j.addbeh.2021.106916. 33798917

[46] Lim CCW; Sun T; Vu G; Chan GCK; Leung J The underbelly of E-cigarette advertising: regulating online markets on social media platforms. Harm Reduction Journal 2024, 21, 105, doi:10.1186/s12954-024-01027-5. 38811969 PMC11134850

[47] Evans W; Andrade E; Pratt M; Mottern A; Chavez S; Calzetta-Raymond A; Gu J Peer-to-Peer Social Media as an Effective Prevention Strategy: Quasi-Experimental Evaluation. JMIR Mhealth Uhealth 2020, 8, e16207, doi:10.2196/16207. 32374270 PMC7240438

[48] Hunter RF; de la Haye K; Murray JM; Badham J; Valente TW; Clarke M; Kee F Social network interventions for health behaviours and outcomes: A systematic review and meta-analysis. PLoS Med 2019, 16, e1002890, doi:10.1371/journal.pmed.1002890. 31479454 PMC6719831

[49] Hennessy EA; Tanner-Smith EE; Finch AJ; Sathe N; Kugley S Recovery schools for improving behavioral and academic outcomes among students in recovery from substance use disorders: a systematic review. Campbell Syst Rev 2018, 14, 1–86, doi:10.4073/csr.2018.9. PMC8428024 37131375

[50] Swinkels LTA; Hoeve M; Ter Harmsel JF; Schoonmade LJ; Dekker JJM; Popma A; van der Pol TM The effectiveness of social network interventions for psychiatric patients: A systematic review and meta-analysis. Clin Psychol Rev 2023, 104, 102321, doi:10.1016/j.cpr.2023.102321. 37499318

[51] Latkin CA; Knowlton AR Social Network Assessments and Interventions for Health Behavior Change: A Critical Review. Behav Med 2015, 41, 90–97, doi:10.1080/08964289.2015.1034645. 26332926 PMC4786366

[52] Webber M; Fendt-Newlin M A review of social participation interventions for people with mental health problems. Soc Psychiatry Psychiatr Epidemiol 2017, 52, 369–380, doi:10.1007/s00127-017-1372-2. 28286914 PMC5380688

[53] Bonar EE; Goldstick JE; Chapman L; Bauermeister JA; Young SD; McAfee J; Walton MA A social media intervention for cannabis use among emerging adults: Randomized controlled trial. Drug Alcohol Depend 2022, 232, 109345, doi:10.1016/j.drugalcdep.2022.109345. 35144238 PMC9549699

[54] Ramos LMC; Delgadillo J; Vélez S; Dauria E; Salas J; Tolou-Shams M Collecting Social Media Information in a Substance Use Intervention Trial With Adolescent Girls With Lifetime Substance Use History: Observational Study. JMIR Form Res 2021, 5, e25405, doi:10.2196/25405. 34505833 PMC8463944

[55] Lotrean LM, Man M, Gavrilescu C, & Florea M (2021). Electronic cigarette use and its relationship with smoking and alcohol and illicit drug consumption among Romanian university students. Medicina, 57(2), 137. 33557228 10.3390/medicina57020137 PMC7913983

[56] Wallace LN, & Roche MJ (2018). Vaping in context: Links among e-cigarette use, social status, and peer influence for college students. Journal of Drug Education, 48(1–2), 36–53.

[57] Graupensperger S, Benson AJ, Bray BC, & Evans MB (2019). Social cohesion and peer acceptance predict student-athletes’ attitudes toward health-risk behaviors: A within-and between-group investigation. Journal of science and medicine in sport, 22(12), 1280–1286. 31349958 10.1016/j.jsams.2019.07.003 PMC6825540

[58] Hefner KR, Sollazzo A, Mullaney S, Coker KL, & Sofuoglu M (2019). E-cigarettes, alcohol use, and mental health: Use and perceptions of e-cigarettes among college students, by alcohol use and mental health status. Addictive behaviors, 91, 12–20. 30396534 10.1016/j.addbeh.2018.10.040 PMC6358487

[59] Rinker DV, Krieger H, & Neighbors C (2016). Social network factors and addictive behaviors among college students. Current addiction reports, 3, 356–367. 28580226 10.1007/s40429-016-0126-7 PMC5450660

[60] Doxbeck CR, & Osberg TM (2021). It’s Not All Smoke and Mirrors: The Role of Social Norms, Alcohol Use, and Pandemic Partying in e-Cigarette Use During COVID-19. Substance Use & Misuse, 56(10), 1551–1558. 10.1080/10826084.2021.1942058 34193015

